# Bridging the gap: neurodevelopmental disorder risks in inborn errors of immunity

**DOI:** 10.1097/ACI.0000000000001036

**Published:** 2024-10-03

**Authors:** Devika Kurup, Amy M. FitzPatrick, Aleksandra Badura, Ines Serra

**Affiliations:** Department of Neuroscience, Erasmus MC, Rotterdam, The Netherlands

**Keywords:** development, inborn errors of immunity, neurodevelopmental disorders, neurological comorbidities, primary immunodeficiencies

## Abstract

**Purpose of review:**

The aim of this review is to examine published reports of neurodevelopmental phenotypes in patients with inborn errors of immunity (IEI). We briefly discuss potential interactions between the immune and the central nervous system and the implications of this crosstalk for current clinical management guidelines.

**Recent findings:**

An increasing number of reports have described neurodevelopmental disorders (NDDs) comorbid with immune-mediated signs. However, the prevalence of this association in IEIs remains unknown.

**Summary:**

IEIs comprise a group of clinically heterogeneous disorders associated with a number of nonimmune comorbidities. Although certain neurological conditions such as microcephaly are recognized as associated features of some IEIs, NDDs are less well described. We reviewed published clinical descriptions of IEIs and found a number of comorbid NDDs in these patients, including autism spectrum disorder (ASD), behavioral deficits, and intellectual disability. Given the lack of uniform assessments for NDDs, we suspect they may be underdiagnosed in IEIs. As NDDs manifest early and can result in life-long cognitive and emotional deficits, which diminish quality of life and increase healthcare utilization, we hope to elucidate relevant pathomechanisms and raise clinician awareness of these comorbidities so appropriate and timely interventions are sought.

## INTRODUCTION

Inborn errors of immunity (IEI) are characterized by increased susceptibility to infections, autoimmunity, atopy, and malignancies. Although each disease is individually rare, IEIs as a group affect between 1 : 1000 and 1 : 5000 people [1^▪^]. IEI research has predominantly focused on the immunological and oncological manifestations, genetic causes, and treatment options, which range from immunoglobulin replacement to hematopoietic stem cell transplant [2,3,8]. In recent years, however, there has been growing interest in multiorgan presentations, with reports describing comorbidities of the central nervous, gastrointestinal, and hepatic systems [1^▪^,^2^].

Neurological signs such as microcephaly and epilepsy are well documented in a few IEIs [3]. However, more subtle, but no less critical, neurological and neurodevelopmental complications are increasingly being found in IEIs, ranging from depression and anxiety to autism, speech delay, and cognitive deficits [4^▪▪^,^5^]. Although mental health has an important and varying impact in IEIs, including on clinical outcomes, the focus of this review will be on neurodevelopmental disorders (NDDs) [6,7].

NDDs are defined as conditions with an onset during the developmental period (typically before school age), characterized by deficits that produce impairments of personal, social, academic, or occupational functioning [8]. In the setting of an IEI, these may be difficult to disambiguate from immune-driven signs, and clinicians may expect resolution once immunological dysfunction is normalized. This, coupled with the heterogeneity of NDD presentations and a paucity of multidisciplinary care, may delay accurate diagnosis and access to support services [9].

Evidence is revealing that many of the genetic variants that cause IEIs also directly modulate brain processes that contribute to the development of comorbid NDDs [10]. The aim of this review is to shed light on the presence of neurodevelopmental presentations in patients with IEIs, discuss the mechanisms that are likely to underlie these comorbidities, and highlight the need for a multidisciplinary team during IEI assessment. 

**Box 1:**
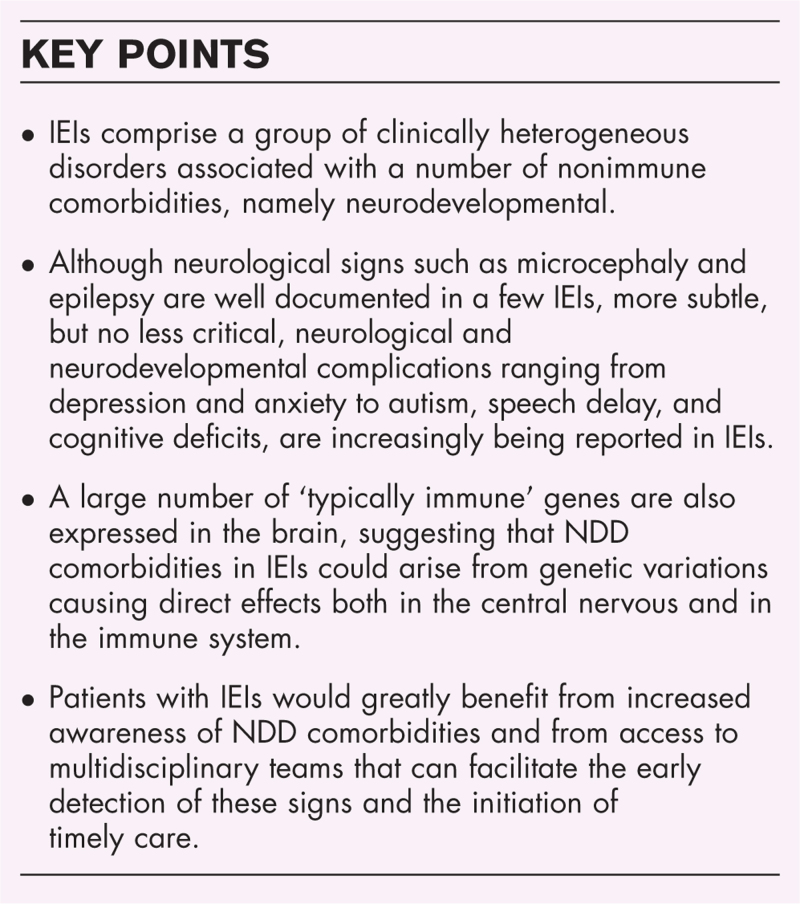
no caption available

## METHODOLOGY

### Literature search

To identify previously reported NDD signs in IEI patients, literature was selected from PubMed, Google Scholar, BASE, and Semantic Scholar using the keywords ‘Neurodevelopmental’, ‘Neurodevelopment’, ‘Neurological’, ‘Autism’, ‘ASD’, ‘Intellectual Disability’ and ‘ID’, and combined with ‘Primary Immune Deficiency’, ‘Primary Immunodeficiency’, ‘PID’, ‘Inborn Errors of Immunity’, and ‘IEI’. After identifying and excluding duplicates, we included a total of 41 publications in the final analysis.

### Presentation categorization

To categorize the neurodevelopmental signs reported in the selected publications, we collected the terms used to describe each disorder. Afterwards, we grouped the identified terms based on the *Diagnostic and Statistical Manual of Mental Disorders*, 5th Edition (DSM-5) [11]. Descriptions of the presence of neurological, neurodevelopmental, and behavioral deficits were then compiled (Table 1) [12]. Patient count was evaluated by reviewing individual data or by calculating it from reported percentages in large cohort studies. When individual cases overlapped with cohort studies, information from the larger study was preferred, unless this did not provide patient counts for signs, in which case individual reports were included instead (Table 2).

**Table 1:** Presentation categorization

	Category	Reported terms and descriptions
Neurodevelopmental disorders		
AD	Attention deficits	Attention problems, ADHD, hyperactivity
ASD	Autism spectrum disorder	Autism, ASD, autism spectrum disorder, inflexibility, rigidity
CgD	Cognitive deficits	Cognitive issues, cognitive problems, cognitive disability
CmD	Communication deficits	Speech, language, communication, social issues, social interaction deficits
ID	Intellectual deficits	Defined as IQ <70 points, mental retardation (DSM-IV diagnosis)
LD	Learning deficits	Learning difficulties (not IQ-related), inability to maintain day-to-day tasks, daily behavior
MD	Movement or motor deficits	Any movement-based disability (could be related to sitting, crawling, head lag, walking, coordination), dysarthria
Neurological and other behavioral deficits		
BD	Behavioral deficits	Behavioral issues not classified as ASD or otherwise unspecified
DD	Developmental delay	Unspecified developmental issues, usually related to growth retardation
DM	Dysmorphism	Microcephaly, facial features, shapes of the head
HI	Hearing impairment	Hearing issues, deafness
SZ	Seizures	Seizures, epilepsy, tremors

Clinical signs were grouped based on the DSM-5 categorization of neurodevelopmental disorder subgroups.

## RESULTS AND DISCUSSION

### Prevalence of neurological complications in Inborn errors of immunity

Neurological manifestations in IEI patients have been recognized for decades, with early reports focusing on complications like meningitis due to opportunistic infections [13]. Subtle neurological and developmental abnormalities were occasionally noted, though not emphasized. Ataxia-telangiectasia, identified in 1941, was among the first IEIs linked to neurological signs namely ataxia, which presents as poor muscle control or clumsy movement [14]. Notably, medical reports may not fully capture the extent of neurological difficulties experienced by patients. A recent study by De Almeida *et al.*[15] aimed to elucidate this by documenting daily neurological complications as reported by patients (*n* = 78) with CVID. Participants reported issues such as changes in speech, vision, memory problems, sleep disturbances, numbness, depression, vertigo, and headaches, illustrating a burden of neurological symptoms that could be addressed with coordinated care.

**Table 2:** Reported neurological and neurodevelopmental presentations in inborn errors of immunity patients

Condition	Gene described in report(s)	Total patient count	Neurodevelopmental disorders							Neurological and cognitive deficits				
			AD	ASD	CmD	CgD	ID	LD	MD	BD	DD	DM	HI	SZ
Activated phosphoinositide 3-kinase delta syndrome (APDS)	*PIK3CD, PIK3R1*	132 [16–20],^a^	–	3	10	9	2	9	2	4	20	5	13	6
Ataxia-telangiectasia (AT)	*ATM*	26 [21,22]	22	–	24	22	23	–	16	–	–	–	–	10
Cohen syndrome	*VPSI3B*	50 [23]	–	9	–	–	21	–	–	–	–	21	–	1
Common variable immunodeficiency disorder (CVID)	*NFKB1*	1 [24]	–	–	1	–	–	1	1	–	1	1	–	–
Hermansky–Pudlak syndrome (HPS)	*AP3D1*	10 [25]	–	–	–	–	–	–	–	–	4	4	7	3
Hyper-lgE syndrome with recurrent infections (HIES)	Undescribed in this report	1 [26]	–	1	1	–	1	–	1	–	1	–	–	–
Immunodeficiency, centromeric instability, and facial anomalies (ICF) syndrome	*DNMT3B, ZBTB24*	38 [27–30]	–	–	26	–	22	1	17	–	1	–	–	4
Immune skeletal dysplasia with neurodevelopmental abnormalities	*EXTL3*	9 [31]	–	–	–	–	*n*	–	*n*	–	*n*	–	–	*n*
Lowry–Wood syndrome (LWS)	*RNU4ATAC*	3 [32]	–	–	1	–	3	–	–	–	–	2	–	–
Nijmegen breakage syndrome (NBS)	*NBS1*	11 [33]	–	–	*n*	–	11	–	–	–	11	11	1	–
Polymerase delta deficiency	*POLD1, POLD2*	2 [34]	1	–	1	–	2	–	1	–	2	1	1	–
Roifman syndrome	*RNU4ATAC*	9 [35–39]	1	–	4	1	5	2	7	1	7	1	–	–
Schimke immuno-osseous dysplasia (SIOD)	*SMARCAL1*	1 [40]	–	–	–	–	–	–	–	–	1	1	–	–
Severe combined immunodeficiency (SCID)	*ADA, PNP, NHEJ1, CORO1A, LIG4*	46 [41–48]	7	–	5	–	21	3	10	–	21	7	2	–
Severe congenital neutropenia (SCN)	*HAX1, RBSN* ^c^	4 [49,50]	–	–	2	–	2	–	1	–	2	–	–	1
Shwachman–Diamond Syndrome (SDS)	*SBDS*	32 [51]	*n*	–	*n*	–	*n*	*n*	–	*n*	*n*	–	–	–
Vici syndrome	*EPG5*	51 [52,53]	–	–	*n*	–	–	–	–	–	51	46	*n*	30
Wiedemann–Steiner syndrome	*KMT2A*	104 [54]	46	22	–	–	*n*	–	–	–	101	*n*	–	21
X-linked agammaglobulinemia (XLA)	*BTK*	3 [55],^b^	–	1	–	–	–	–	–	–	–	–	3	–
X-linked immunodeficiency with magnesium defect (XMEN)	*MAGT1*	3 [56]	–	–	–	–	2	–	–	1	–	–	–	–

Summary of patient count with reported neurological and neurodevelopmental presentations across various IEIs, defined using the International Union of Immunological Societies (IUIS) Expert Committee classification [3]. –, no data reported; AD, attention deficits; ASD, autism spectrum disorder; BD, behavioral deficits; CmD, communication deficits; CgD, cognitive deficits; DD, developmental delay; DM, dysmorphism; HI, hearing impairment; ID, intellectual deficits; LD, learning deficits; MD, movement or motor deficits; *n*, reported but with unspecified patient numbers; SZ, seizures.

a One patient carried an APDS variant, and a Smith–Magenis syndrome (SMS) variant in the *RAI1* gene.

b Two-thirds of patients had genetic variants in addition to *BTK*, namely in *TIMM8A*, *TAF7L*, and *DRP2*.

c Gene currently not recognized as IEI causing.

### Neurodevelopmental disorder presentations in Inborn errors of immunity

Most of the reported patients with IEIs and NDDs presented with APDS, Wiedemann–Steiner syndrome (WSS), immunodeficiency with centromeric instability and facial anomalies (ICF), ataxia-telangiectasia, and Vici syndrome. Regarding WSS, a review by Sheppard *et al*. [54] found that nearly 97% of 104 individuals had developmental deficits or intellectual disability. Additionally, 44% exhibited hyperactivity, 33% displayed aggressive behavior, and 21% were diagnosed with ASD. In ataxia-telangiectasia, patients at early and late stages of disease presented with below average results when tested for verbal IQ, comprehension and vocabulary, visuospatial skills, and working memory [19]. Late-stage patients had additional impairments in attention, working memory, and abstract reasoning, with lower comprehension scores than early-stage patients. This progression underscores the broad spectrum of cognitive and developmental challenges associated with ataxia-telangiectasia, as well as the progressive nature of comorbid NDDs in this disease.

In ICF, one study observed intellectual disability in most patients with speech delays noted in virtually all, although descriptions of motor delays are also common [27–30]. A study on Vici syndrome, which is well known to be a multisystem disorder, reported gross developmental delays in all 50 study patients, particularly noting the presence of delayed social and communication skills [52,53]. Finally, NDD presentations have also been relatively common in APDS, with cohort studies reporting developmental delay associated with speech and learning deficits in 20–30% of patients [16,20]. Eickholt *et al.*[57] demonstrated that a protein product mutated in APDS, Pik3cd, was strongly expressed in the mouse's developing CNS, providing a putative link between brain and immunological deficits. Of note, Serra and colleagues described a patient with APDS who underperformed on a series of visuomotor tasks, despite normalized immune function and lack of apparent NDD symptomatology [15], further suggesting that altered brain function in IEIs can putatively arise from immune-independent mechanisms.

### Underlying mechanisms of neuroimmunological interaction

For several IEIs, delays in early developmental milestones, including crawling, talking, or walking, have been reported [4^▪▪^,^19^,^21^,^28^,^33^,^58^,^59^]. Physiologically, proteins considered to be strictly immune-related are expressed in the brain and participate in a number of processes essential for typical development including neurogenesis, cell migration, and synapse formation and remodeling [60]. For example, complement component 1q, whose deficiency leads to IEI, affects neuronal translation mechanisms in an age-dependent manner [61], whereas MHC class I molecules, that function as antigen-presenting receptors in the immune system, are expressed in the developing human cerebral cortex and regulate dendritic spine morphology and total number of synapses [62,63]. Dysregulation of some of these ‘typically immune’ genes has also been found in people with NDDs [64,65]. Transcriptomic studies in postmortem brains of people with ASD revealed broad dysregulation of multiple immune response genes [66], with large-scale GWAS studies, identifying associations of immune genes with specific autistic traits like ‘attention to detail’ and ‘social skills’ [67].

Microglia, the resident immune cells of the brain, also mediate interactions between the immune system and CNS. Unlike neurons, which derive from neuronal progenitor cells, microglia originate from the hematopoietic lineage. From the fourth postconception week, microglia leave the periphery and colonize the developing brain, where they regulate the production of neural precursor cells and the formation of brain circuitries [68]. Maternal infections, which are thought to increase the risk of autism [69], were shown to disrupt these processes, triggering abnormal cytokine production and the activation of inflammatory pathways during development [70].

These overlaps suggest that NDD comorbidities in IEIs could arise from genetic variations causing direct effects both in the CNS and immune system, in addition to secondary effects resulting from immune dysregulation and chronic disease. Because neurodevelopmental symptoms carry a large disease burden, negatively impact the quality of life of the affected individuals [71], and can be progressive, there is a growing urgency to understand the actual pathomechanisms of these comorbidities in IEIs.

### Future directions for neurodevelopmental disorder assessments in inborn errors of immunity

Despite the lack of systematic neurodevelopmental assessments in IEI, available evidence suggests that NDDs might be more common in these patients than in the general population. This, together with the already described neurological signs and other features, calls for a concerted effort towards the inclusion of more formal neuropsychiatric evaluations at least in some IEI subgroups.

Though diagnostic delays are reducing, IEI diagnoses remain variable between countries and significant delays in diagnoses persist worldwide [72^▪^,^73^,^74^]. Nonetheless, it is estimated that around 2/3 of IEI patients are diagnosed during pediatric ages and that roughly 1/5 are diagnosed before the age of 4 [75]. This time period represents a crucial window of opportunity to identify NDDs, with the hope that early intervention may prevent progressive dysfunction and maximize therapeutic effectiveness [76]. Thus, early IEI diagnoses not only lessen the personal, disease and economic burden associated with immune dysfunction but may positively affect neurodevelopmental trajectories [73].

Providing added neuropsychiatric care to IEI patients should not come at the cost of unnecessary additional pressure in the medical system and, therefore, patient selection is paramount. We posit Table 2 could be used to identify patients who could benefit from a neuropsychological evaluation. Additionally, the International Union of Immunological Societies (IUIS) classification of IEIs already acknowledges the presence of a large group of conditions and mutations that are associated with increased reporting of NDD features (category 2: combined immunodeficiencies with associated or syndromic features). We argue that patients who meet clinical or genetic criteria for category 2 disorders should be preferentially referred for a formal neuropsychiatric evaluation, especially during pediatric ages and despite the possible absence of noticeable NDD symptomatology [76]. Finally, NDDs can also be a significant barrier to the pediatric to adult transition of care in IEI patients, highlighting once more the relevance of these phenotypes for overall patient management and outcome [77–79].

### Limitations

The terminology used for the search, although accurate, may not be fully representative of how clinical immunologists tracked neurodevelopmental patient signs in literature. For example, a clinician may have noted a child presented with ‘trouble with school’. This presentation could actually encompass many neurodevelopmental issues while lacking the word ‘neurodevelopment’ or other DSM-V diagnoses. Furthermore, these signs may not be captured in immunological literature at all. For these reasons, we think the prevalence of NDDs in IEIs herein are appreciably low, especially as IEIs with known cognitive deficits such as DiGeorge syndrome, Jacobsen syndrome, and others were not returned in the results. A systematic review using additional terminology could attempt to improve these results; however, we posit that the need for coordinated care and collaborative, interdepartmental investigations, and a move toward unified terminology are the main recommendations of this work.

## CONCLUSION

Although individually rare, collectively, IEIs affect a significant number of people. NDDs within these IEIs significantly affect the quality of life and independence of patients, and pose considerable burden to healthcare systems. Although the prevalence and distribution of NDDs in IEIs require further investigation, patients would benefit, firstly, from increased awareness of this comorbidity and, secondly, from access to multidisciplinary teams that can facilitate their early detection and care. Intervention timing matters because the brief window of childhood neurodevelopment sets the foundation for lifelong cognition and emotional regulation.

## Acknowledgements

*We thank Virgil Dalm for constructive discussions regarding the subject of this review.*

### Financial support and sponsorship

*This work was supported by the Netherlands Organization for Scientific Research (NWO), the NWO NWA-ORC 2022 SCANNER grant, the Erasmus MC Convergence Health and Technology Integrative Neuromedicine Flagship Program 2022 (A.B. and I.S.) and the Horizon 2020 Research and Innovation programme, MSCA-ITN PIPgen #955534 (A.B and D.K.).*

### Conflicts of interest

*There are no conflicts of interest.*

## REFERENCES AND RECOMMENDED READING

Papers of particular interest, published within the annual period of review, have been highlighted as:

▪ of special interest
▪▪ of outstanding interest

## References

[1▪] Akalu YT Bogunovic D. Inborn errors of immunity: an expanding universe of disease and genetic architecture. Nat Rev Genet 2024; 25:184–195. 37863939 10.1038/s41576-023-00656-z PMC13489759

[2] Resnick ES Moshier EL Godbold JH Cunningham-Rundles C. Morbidity and mortality in common variable immune deficiency over 4 decades. Blood 2012; 119:1650–1657. 22180439 10.1182/blood-2011-09-377945 PMC3286343

[3] Tangye SG Al-Herz W Bousfiha A. Human inborn errors of immunity: 2022 update on the classification from the International Union of Immunological Societies Expert Committee. J Clin Immunol 2022; 42:1473–1507. 35748970 10.1007/s10875-022-01289-3 PMC9244088

[4▪▪] Kose H Karali Z Bodur M. Neurological involvement in patients with primary immunodeficiency. Allergol Immunopathol 2024; 52:85–92. 10.15586/aei.v52i1.961 38186198

[5] Manusama OR van Beveren NJM van Hagen PM. Psychological symptoms in primary immunodeficiencies: a common comorbidity? J Clin Immunol 2022; 42:695–698. 35043302 10.1007/s10875-022-01207-7 PMC9016014

[6] Nicholson B Goodman R Day J. Quality of life and social and psychological outcomes in adulthood following allogeneic HSCT in childhood for inborn errors of immunity. J Clin Immunol 2022; 42:1451–1460. 35723794 10.1007/s10875-022-01286-6 PMC9674756

[7] Routes J Costa-Carvalho BT Grimbacher B. Health-related quality of life and health resource utilization in patients with primary immunodeficiency disease prior to and following 12 months of immunoglobulin G treatment. J Clin Immunol 2016; 36:450–461. 27091140 10.1007/s10875-016-0279-0 PMC4896988

[8] Diagnostic and Statistical Manual of Mental Disorders: DSM-5 (5th edition). Ref Rev 2014; 28:36–37.

[9] Boulton KA Hodge M-A Jewell A. Original research: diagnostic delay in children with neurodevelopmental conditions attending a publicly funded developmental assessment service: findings from the Sydney Child Neurodevelopment Research Registry. BMJ Open 2023; 13:e069500. 10.1136/bmjopen-2022-069500 PMC9896183 36725093

[10] Boulanger LM. Immune proteins in brain development and synaptic plasticity. Neuron 2009; 64:93–109. 19840552 10.1016/j.neuron.2009.09.001

[11] American Psychiatric Association: *Diagnostic and statistical manual of mental disorders: DSM-5TM*. American Psychiatric Association; 1980.

[12] Buie T Margolis K. Considerations for treating autistic individuals in gastroenterology clinics. Lancet Gastroenterol Hepatol 2024; 9:684–686. 38823399 10.1016/S2468-1253(24)00153-5

[13] Noh LM Low SM Lajin I Abdullah N. Antibody deficiency with hyper IgM--a case report. Malays J Pathol 1992; 14:121–123. 1304625

[14] Teive HAG Moro A Moscovich M. Ataxia-telangiectasia - a historical review and a proposal for a new designation: ATM syndrome. J Neurol Sci 2015; 355:3–6. 26050521 10.1016/j.jns.2015.05.022 PMC5161405

[15] De Almeida BI Smith TL Delic A. Neurologic manifestations of common variable immunodeficiency: impact on quality of life. Neurol Neuroimmunol Neuroinflamm 2023; 10:e200088. 36797058 10.1212/NXI.0000000000200088 PMC9936420

[16] Elkaim E Neven B Bruneau J. Clinical and immunologic phenotype associated with activated phosphoinositide 3-kinase δ syndrome 2: a cohort study. J Allergy Clin Immunol 2016; 138:210.e9–218.e9. 27221134 10.1016/j.jaci.2016.03.022

[17] Serra I Manusama OR Kaiser FMP. Activated PI3Kδ syndrome, an immunodeficiency disorder, leads to sensorimotor deficits recapitulated in a murine model. Brain Behav Immun Health 2021; 18:100377. 34786564 10.1016/j.bbih.2021.100377 PMC8579111

[18] Moreno-Corona N Chentout L Poggi L. Two monogenetic disorders, activated PI3-kinase-δ syndrome 2 and Smith-Magenis syndrome, in one patient: case report and a literature review of neurodevelopmental impact in primary immunodeficiencies associated with disturbed PI3K signaling. Front Pediatr 2021; 9:688022. 34249818 10.3389/fped.2021.688022 PMC8266209

[19] Oh J Garabedian E Fuleihan R Cunningham-Rundles C. Clinical manifestations and outcomes of activated phosphoinositide 3-kinase δ syndrome from the USIDNET cohort. J Allergy Clin Immunol Pract 2021; 9:4095–4102. 34352450 10.1016/j.jaip.2021.07.044 PMC8578310

[20] Coulter TI Chandra A Bacon CM. Clinical spectrum and features of activated phosphoinositide 3-kinase δ syndrome: a large patient cohort study. J Allergy Clin Immunol 2017; 139:597.e4–606.e4. 27555459 10.1016/j.jaci.2016.06.021 PMC5292996

[21] Lanzi G Balottin U Franciotta D. Clinical, cytogenetic and immunological aspects in 4 cases resembling ataxia telangiectasia. Eur Neurol 1992; 32:121–125. 1375558 10.1159/000116807

[22] Hoche F Frankenberg E Rambow J. Cognitive phenotype in ataxia-telangiectasia. Pediatr Neurol 2014; 51:297–310. 25037873 10.1016/j.pediatrneurol.2014.04.027

[23] Zorn M Kühnisch J Bachmann S Seifert W. Disease relevance of rare VPS13B missense variants for neurodevelopmental Cohen syndrome. Sci Rep 2022; 12:9686. 35690661 10.1038/s41598-022-13717-w PMC9188546

[24] Franco-Jarava C Valenzuela I Riviere JG. Common variable immunodeficiency and neurodevelopmental delay due to a 13Mb deletion on chromosome 4 including the NFKB1 gene: a case report. Front Immunol 2022; 13:897975. 35784294 10.3389/fimmu.2022.897975 PMC9247144

[25] Frohne A Koenighofer M Cetin H. A homozygous AP3D1 missense variant in patients with sensorineural hearing loss as the leading manifestation. Hum Genet 2023; 142:1077–1089. 36445457 10.1007/s00439-022-02506-0 PMC10449960

[26] Grimbacher B Dutra AS Holland SM. Analphoid marker chromosome in a patient with hyper-IgE syndrome, autism, and mild mental retardation. Genet Med 1999; 1:213–218. 11256675 10.1097/00125817-199907000-00008

[27] Hagleitner MM Lankester A Maraschio P. Clinical spectrum of immunodeficiency, centromeric instability and facial dysmorphism (ICF syndrome). J Med Genet 2008; 45:93–99. 17893117 10.1136/jmg.2007.053397

[28] Weemaes CMR van Tol MJD Wang J. Heterogeneous clinical presentation in ICF syndrome: correlation with underlying gene defects. Eur J Hum Genet 2013; 21:1219–1225. 23486536 10.1038/ejhg.2013.40 PMC3798845

[29] Aminorroaya A Rayzan E Shahkarami S. Novel DNMT3B mutation in a patient with immunodeficiency, centromeric instability, and facial anomalies (ICF) syndrome and a bronchopulmonary collateral artery. Endocr Metab Immune Disord Drug Targets 2023; 23:410–415. 35996251 10.2174/1871530322666220822141722

[30] Cerbone M Wang J Van der Maarel SM. Immunodeficiency, centromeric instability, facial anomalies (ICF) syndrome, due to ZBTB24 mutations, presenting with large cerebral cyst. Am J Med Genet A 2012; 158A:2043–2046. 22786748 10.1002/ajmg.a.35486 PMC3402683

[31] Oud MM Tuijnenburg P Hempel M. Mutations in EXTL3 cause neuro-immuno-skeletal dysplasia syndrome. Am J Hum Genet 2017; 100:281–296. 28132690 10.1016/j.ajhg.2017.01.013 PMC5294674

[32] Farach LS Little ME Duker AL. The expanding phenotype of RNU4ATAC pathogenic variants to Lowry Wood syndrome. Am J Med Genet A 2018; 176:465–469. 29265708 10.1002/ajmg.a.38581 PMC6774248

[33] Chrzanowska KH Kleijer WJ Krajewska-Walasek M. Eleven Polish patients with microcephaly, immunodeficiency, and chromosomal instability: the Nijmegen breakage syndrome. Am J Med Genet 1995; 57:462–471. 7545870 10.1002/ajmg.1320570321

[34] Conde CD Petronczki ÖY Baris S. Polymerase δ deficiency causes syndromic immunodeficiency with replicative stress. J Clin Invest 2019; 129:4194–4206. 31449058 10.1172/JCI128903 PMC6763221

[35] Mandel K Grunebaum E Benson L. Noncompaction of the myocardium associated with Roifman syndrome. Cardiol Young 2001; 11:240–243. 11293748 10.1017/s1047951101000208

[36] Robertson SP Rodda C Bankier A. Hypogonadotrophic hypogonadism in Roifman syndrome. Clin Genet 2000; 57:435–438. 10905663 10.1034/j.1399-0004.2000.570606.x

[37] de Vries PJ McCartney DL McCartney E. The cognitive and behavioural phenotype of Roifman syndrome. J Intellect Disabil Res 2006; 50:690–696. 16901296 10.1111/j.1365-2788.2006.00817.x

[38] Clifford D Moloney F Leahy TR Murray DM. Roifman syndrome: a description of further immunological and radiological features. BMJ Case Rep 2022; 15:e249109. 10.1136/bcr-2022-249109 PMC9024203 35450878

[39] Roifman CM. Antibody deficiency, growth retardation, spondyloepiphyseal dysplasia and retinal dystrophy: a novel syndrome. Clin Genet 1999; 55:103–109. 10189087 10.1034/j.1399-0004.1999.550206.x

[40] Simon AJ Lev A Jeison M. Novel SMARCAL1 bi-allelic mutations associated with a chromosomal breakage phenotype in a severe SIOD patient. J Clin Immunol 2014; 34:76–83. 24197801 10.1007/s10875-013-9957-3

[41] Sauer AV Hernandez RJ Fumagalli F. Alterations in the brain adenosine metabolism cause behavioral and neurological impairment in ADA-deficient mice and patients. Sci Rep 2017; 7:40136. 28074903 10.1038/srep40136 PMC5225479

[42] Cagdas D Gur Cetinkaya P Karaatmaca B. ADA deficiency: evaluation of the clinical and laboratory features and the outcome. J Clin Immunol 2018; 38:484–493. 29744787 10.1007/s10875-018-0496-9

[43] Frizinsky S Rechavi E Barel O. Novel NHEJ1 pathogenic variant linked to severe combined immunodeficiency, microcephaly, and abnormal T and B cell receptor repertoires. Front Pediatr 2022; 10:883173. 35967585 10.3389/fped.2022.883173 PMC9363661

[44] Shiow LR Paris K Akana MC. Severe combined immunodeficiency (SCID) and attention deficit hyperactivity disorder (ADHD) associated with a Coronin-1A mutation and a chromosome 16p11.2 deletion. Clin Immunol 2009; 131:24–30. 19097825 10.1016/j.clim.2008.11.002 PMC2692687

[45] O’Driscoll M Cerosaletti KM Girard PM. DNA ligase IV mutations identified in patients exhibiting developmental delay and immunodeficiency. Mol Cell 2001; 8:1175–1185. 11779494 10.1016/s1097-2765(01)00408-7

[46] Al-Saud B Al Alawi Z Hussain FB. A case with purine nucleoside phosphorylase deficiency suffering from late-onset systemic lupus erythematosus and lymphoma. J Clin Immunol 2020; 40:833–839. 32514656 10.1007/s10875-020-00800-y

[47] Recio MJ Dominguez-Pinilla N Perrig MS. Extreme phenotypes with identical mutations: two patients with same nonsense NHEJ1 homozygous mutation. Front Immunol 2018; 9:2959. 30666249 10.3389/fimmu.2018.02959 PMC6330288

[48] Nofech-Mozes Y Blaser SI Kobayashi J. Neurologic abnormalities in patients with adenosine deaminase deficiency. Pediatr Neurol 2007; 37:218–221. 17765813 10.1016/j.pediatrneurol.2007.03.011

[49] Rezaei N Chavoshzadeh Z Alaei R. Association of HAX1 deficiency with neurological disorder. Neuropediatrics 2007; 38:261–263. 18330843 10.1055/s-2008-1062704

[50] Magoulas PL Shchelochkov OA Bainbridge MN. Syndromic congenital myelofibrosis associated with a loss-of-function variant in RBSN. Blood 2018; 132:658–662. 29784638 10.1182/blood-2017-12-824433 PMC6085991

[51] Kerr EN Ellis L Dupuis A. The behavioral phenotype of school-age children with Shwachman Diamond syndrome indicates neurocognitive dysfunction with loss of Shwachman-Bodian-Diamond syndrome gene function. J Pediatr 2010; 156:433.e1–438.e1. 19906387 10.1016/j.jpeds.2009.09.026

[52] Byrne S Jansen L U-King-Im J-M. EPG5-related Vici syndrome: a paradigm of neurodevelopmental disorders with defective autophagy. Brain 2016; 139:765–781. 26917586 10.1093/brain/awv393 PMC4766378

[53] Balasubramaniam S Riley LG Vasudevan A. EPG5-related Vici syndrome: a primary defect of autophagic regulation with an emerging phenotype overlapping with mitochondrial disorders. JIMD Rep 2018; 42:19–29. 29159459 10.1007/8904_2017_71 PMC6226401

[54] Sheppard SE Campbell IM Harr MH. Expanding the genotypic and phenotypic spectrum in a diverse cohort of 104 individuals with Wiedemann-Steiner syndrome. Am J Med Genet A 2021; 185:1649–1665. 33783954 10.1002/ajmg.a.62124 PMC8631250

[55] Arai T Zhao M Kanegane H. Genetic analysis of contiguous X-chromosome deletion syndrome encompassing the BTK and TIMM8A genes. J Hum Genet 2011; 56:577–582. 21753765 10.1038/jhg.2011.61

[56] Blommaert E Péanne R Cherepanova NA. Mutations in MAGT1 lead to a glycosylation disorder with a variable phenotype. Proc Natl Acad Sci U S A 2019; 116:9865–9870. 31036665 10.1073/pnas.1817815116 PMC6525510

[57] Eickholt BJ Ahmed AI Davies M. Control of axonal growth and regeneration of sensory neurons by the p110delta PI 3-kinase. PLoS One 2007; 2:e869. 17846664 10.1371/journal.pone.0000869 PMC1959241

[58] Saettini F Herriot R Prada E. Prevalence of immunological defects in a cohort of 97 Rubinstein-Taybi syndrome patients. J Clin Immunol 2020; 40:851–860. 32594341 10.1007/s10875-020-00808-4

[59] Yu Y Jia X Yin H. A novel variant in BCL11B in an individual with neurodevelopmental delay: a case report. Mol Genet Genomic Med 2023; 11:e2132. 36683525 10.1002/mgg3.2132 PMC10094078

[60] Garay PA Kimberley McAllister A. Novel roles for immune molecules in neural development: implications for neurodevelopmental disorders. Front Synaptic Neurosci 2010; 2:136. 21423522 10.3389/fnsyn.2010.00136 PMC3059681

[61] Scott-Hewitt N Mahoney M Huang Y. Microglial-derived C1q integrates into neuronal ribonucleoprotein complexes and impacts protein homeostasis in the aging brain. Cell 2024; 187:4193.e24–4212.e24. 38942014 10.1016/j.cell.2024.05.058 PMC12344723

[62] Zhang A Yu H He Y. Developmental expression and localization of MHC class I molecules in the human central nervous system. Exp Brain Res 2015; 233:2733–2743. 26169100 10.1007/s00221-015-4345-2

[63] Lazarczyk MJ Eyford BA Varghese M. The intracellular domain of major histocompatibility class-I proteins is essential for maintaining excitatory spine density and synaptic ultrastructure in the brain. Sci Rep 2023; 13:6448. 37081001 10.1038/s41598-023-30054-8 PMC10119172

[64] Kaiser FMP Gruenbacher S Oyaga MR. Biallelic PAX5 mutations cause hypogammaglobulinemia, sensorimotor deficits, and autism spectrum disorder. J Exp Med 2022; 219:e20220498. 35947077 10.1084/jem.20220498 PMC9372349

[65] Gofin Y Wang T Gillentine MA. Delineation of a novel neurodevelopmental syndrome associated with PAX5 haploinsufficiency. Hum Mutat 2022; 43:461–470. 35094443 10.1002/humu.24332 PMC8960338

[66] Gandal MJ Haney JR Wamsley B. Broad transcriptomic dysregulation occurs across the cerebral cortex in ASD. Nature 2022; 611:532–539. 36323788 10.1038/s41586-022-05377-7 PMC9668748

[67] Arenella M Cadby G De Witte W. Potential role for immune-related genes in autism spectrum disorders: evidence from genome-wide association meta-analysis of autistic traits. Autism 2022; 26:361–372. 34344231 10.1177/13623613211019547 PMC8814945

[68] Menassa DA Muntslag TAO Martin-Estebané M. The spatiotemporal dynamics of microglia across the human lifespan. Dev Cell 2022; 57:2127.e6–2139.e6. 35977545 10.1016/j.devcel.2022.07.015 PMC9616795

[69] Nudel R Thompson WK Børglum AD. Maternal pregnancy-related infections and autism spectrum disorder—the genetic perspective. Transl Psychiatry 2022; 12:334. 35974006 10.1038/s41398-022-02068-9 PMC9381559

[70] Loayza M Lin S Carter K. Maternal immune activation alters fetal and neonatal microglia phenotype and disrupts neurogenesis in mice. Pediatr Res 2023; 93:1216–1225. 35963885 10.1038/s41390-022-02239-w

[71] GBD 2016 Neurology Collaborators. Global, regional, and national burden of neurological disorders, 1990–2016: a systematic analysis for the Global Burden of Disease Study. Lancet Neurol 2019; 18:459–480. 30879893 10.1016/S1474-4422(18)30499-X PMC6459001

[72▪] Lawrence M Rider N Cunningham-Rundles C. Disparities in diagnosis, access to specialist care, and treatment for inborn errors of immunity. J Allergy Clin Immunol Pract 2024; 12:282–287. 10.1016/j.jaip.2023.10.041 39492552

[73] Anderson JT Cowan J Condino-Neto A. Health-related quality of life in primary immunodeficiencies: Impact of delayed diagnosis and treatment burden. Clin Immunol 2022; 236:108931. 35063670 10.1016/j.clim.2022.108931

[74] Chong-Neto HJ Radwan N Condino-Neto A. Newborn screening for inborn errors of immunity: the status worldwide. World Allergy Organ J 2024; 17:100920. 38974948 10.1016/j.waojou.2024.100920 PMC11225001

[75] Quinn J Modell V Orange JS Modell F. Growth in diagnosis and treatment of primary immunodeficiency within the global Jeffrey Modell Centers Network. Allergy Asthma Clin Immunol 2022; 18:19. 35246253 10.1186/s13223-022-00662-6 PMC8896271

[76] Veenstra-VanderWeele J Warren Z. Intervention in the context of development: pathways toward new treatments. Neuropsychopharmacology 2014; 40:225–237. 25182180 10.1038/npp.2014.232 PMC4262912

[77] Sanchez LA Tang M Ahmed A. Transition of care in inborn errors of immunity: outcomes of a single-center quality improvement initiative. J Allergy Clin Immunol Pract 2023; 11:2245. 37119980 10.1016/j.jaip.2023.04.024 PMC11142330

[78] Tadros S Burns SO. Transition of care in inborn errors of immunity. Curr Opin Allergy Clin Immunol 2023; 23:455–460. 37797181 10.1097/ACI.0000000000000948 PMC10621636

[79] Mejía González MA Quijada Morales P Escobar MÁ. Navigating the transition of care in patients with inborn errors of immunity: a single-center's descriptive experience. Front Immunol 2023; 14:1263349. 37854610 10.3389/fimmu.2023.1263349 PMC10579936

